# A Novel Adenosine Kinase from *Bombyx mori*: Enzymatic Activity, Structure, and Biological Function

**DOI:** 10.3390/ijms20153732

**Published:** 2019-07-31

**Authors:** Kai Song, Yu Li, Huawei He, Lina Liu, Ping Zhao, Qingyou Xia, Yejing Wang

**Affiliations:** 1 State Key Laboratory of Silkworm Genome Biology, Biological Science Research Center, Southwest University, Beibei, Chongqing 400715, China; 2 College of Biotechnology, Southwest University, Beibei, Chongqing 400715, China; 3 Chongqing Key Laboratory of Sericultural Science, Chongqing Engineering and Technology Research Center for Novel Silk Materials, Southwest University, Beibei, Chongqing 400715, China

**Keywords:** adenosine kinase, *Bombyx mori*, enzymatic activity, structure

## Abstract

Adenosine kinase (ADK) is the first enzyme in the adenosine remediation pathway that catalyzes adenosine phosphorylation into adenosine monophosphate, thus regulating adenosine homeostasis in cells. To obtain new insights into ADK from *Bombyx mori* (BmADK), we obtained recombinant BmADK, and analyzed its activity, structure, and function. Gel-filtration showed BmADK was a monomer with molecular weight of approximately 38 kDa. Circular dichroism spectra indicated BmADK had 36.8% α-helix and 29.9% β-strand structures, respectively. The structure of BmADK was stable in pH 5.0–11.0, and not affected under 30 °C. The melting temperature and the enthalpy and entropy changes in the thermal transition of BmADK were 46.51 ± 0.50 °C, 253.43 ± 0.20 KJ/mol, and 0.79 ± 0.01 KJ/(mol·K), respectively. Site-directed mutagenesis demonstrated G68, S201, E229, and D303 were key amino acids for BmADK structure and activity. In particular, S201A mutation significantly increased the α-helix content of BmADK and its activity. BmADK was located in the cytoplasm and highly expressed in the silk gland during the pre-pupal stage. RNA interference revealed the downregulation of *BmADK* decreased *ATG-8*, *Caspase-9*, *Ec-R*, *E74A*, and *Br-C* expression, indicating it was likely involved in 20E signaling, apoptosis, and autophagy to regulate silk gland degeneration and silkworm metamorphosis. Our study greatly expanded the knowledge on the activity, structure, and role of ADK.

## 1. Introduction

Adenosine kinase (EC 2.7.1.20, ADK) is the most important enzyme in adenosine metabolism. It is one of the most common and abundant nucleoside kinases [1,2]. The protein sequence of ADK is conserved among several eukaryotic species [3,4]. ADK is the first enzyme in the adenosine remediation pathway, and an important regulator of extracellular adenosine concentration [5], which has been well elucidated in animal models of epilepsy and ischemia [6,7]. ADK was first isolated from yeast in 1951. In the past decades, ADK properties including substrate specificity [8], inhibitors [9], metal ions [10], ATP, and inorganic phosphate [11] have been extensively investigated.

In 1998, the first crystal structure of human ADK was resolved [12]. ADK contains two domains, one being a large α/β domain and the other, a small cap domain. The large domain is composed of nine β-strands and eight α-helices, providing an appropriate environment for the specific binding of ATP and adenosine. The small domain is composed of five β-strands and two α-helices acting as a lid on the top of the activation center. The domains are connected by four peptides, and adenosine as the substrate binds in the cleft formed by the two domains. The nucleotide binding sites are located in the groove of the catalytic domain [12]. In 2000, the crystal structure of *Toxoplasma gondii* adenosine kinase revealed a different catalytic mechanism which involves a rotation of the catalytic domain and the formation of an anion hole mediated by ATP binding and a conformational change from α-helix to random coil [13]. Compared to human ADK, the ADK of *Mycobacterium tuberculosis* (Mt) is greatly different [11,14]. Although both of them are composed of a large domain and a small lid-like domain, MtADK shares less than 20% sequence identity with that of human ADK. The key difference between MtADK and human ADK is that MtADK is a functional homodimer, but the active formation of human ADK is a monomer [12,14].

The function of ADK is to maintain the balance of adenosine concentration in cells by phosphorylation of adenosine. As a result, any change in enzymatic activity can trigger a large number of physiological diseases. In transgenic mice lacking ADK, the newborn mice have abnormal temperature control, and deaths occur on the fourth day. On the eighth day, the mortality rate is as high as 88%. The livers of the dead mice display obvious lesions, and the content of intermediate products in methyl transfer reactions such as *S*-adenosylhomocysteine and *S*-adenosylmethionine increases, indicating that ADK is involved in the methylation reaction pathway [15]. ADK activity is reduced by 40–50% in diabetes models, and its transcription level is also reduced by 50%. However, the recovery of ADK activity promotes the proliferation of t-lymphocytes [16]. The inhibition of ADK activity by specific inhibitors significantly prevents the occurrence of inflammation [17], and promotes the growth of liver cancer cells, whereas the activation of ADK expression promotes the growth of colorectal cancer cells [18].

*Bombyx mori* is a kind of *Lepidoptera* model insect with high economic value. Although ADK has been extensively studied for decades, the biochemical properties, structure, and biological role of ADK from the silkworm (BmADK) are largely unknown. Here, we expressed and purified the recombinant BmADK protein and its variants in vitro, and studied its activity, structure, cellular localization, and potential roles in the silkworm. Our study advanced a better understanding of the enzymatic activity, structure, and biological role of ADK.

## 2. Results

### 2.1. Bioinformatic Analysis, Expression, and Purification of BmADK

**Figure 1:**
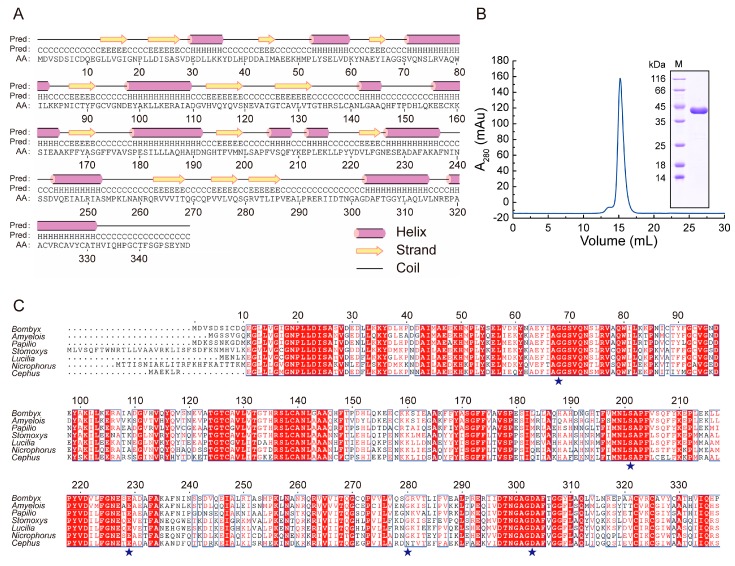
Structural prediction, purification of heterologously expressed *Bombyx mori* adenosine kinase (BmADK), and sequence comparison of ADK from different species. (**A**) Diagram shows the predicted secondary structure of BmADK. (**B**) Purification of BmADK by gel filtration and SDS-PAGE analysis. (**C**) Multiple sequence alignments of ADKs from *Bombyx mori*, *Amyelois transitella*, *Papilio troilus*, *Stomoxys calcitrans*, *Lucilia cuprina*, *Nicrophorus orbicollis*, and *Cephus cinctus*. Similarities are highlighted in red and mutation sites are marked with blue stars.

To characterize the properties and structure of BmADK, we carried out a bioinformatic analysis. The result showed that BmADK is composed of 1050 bp and encodes 349 amino acids. Its theoretical pI was predicted to be 5.13, and the theoretical molecular weight was 38 kDa. Secondary structure prediction showed that BmADK contained a large number of α-helix and β-strand structures (Figure 1A).

BmADK was purified after heterologous expression by Ni-NTA affinity column and gel filtration chromatography. The molecular weight of BmADK was estimated by SDS-PAGE and volume exclusion chromatography to be approximately 38 kDa, indicating that BmADK existed as a monomer in the buffer (Figure 1B). BmADK mutants were purified by the same method as the wild-type (WT) BmADK. As shown in Figure S1 (Supplementary Materials), all the mutants existed as monomers in the buffer, indicating that the mutations did not change the state of BmADK in solution.

Multiple sequence alignment was performed to show evolutionarily or structurally related positions between BmADK and its homologs using the sequence of BmADK as the reference. Figure 1C showed that ADKs from different species had an amino acid identity of 37%, especially the GG switch (Gly68 and Gly69), ATP-binding motif NxxE, and catalytic activity center Asp303. To further determine the effect of these conserved amino acids on BmADK activity, we selected five amino acid residues for the mutation tests (Figure 1C). The mutants were G68A, S201A, S201R, S201D, E229A, E229R, G280A, G280D, and D303A, respectively.

### 2.2. Secondary Structure of BmADK

**Figure 2:**
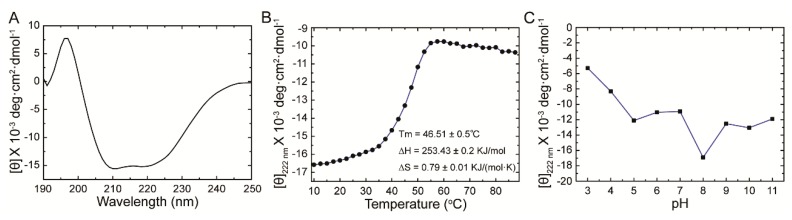
Secondary structure and stability analysis of BmADK. (**A**) CD spectra of BmADK. (**B**) Structural changes of BmADK induced by temperature. (**C**) Structural changes of BmADK in pH range of 3.0–11.0. The mean residue ellipticities at 222 nm are used to represent the structural changes of BmADK induced by pH and temperature.

The secondary structure of BmADK was measured by circular dichroism (CD) spectroscopy. As shown in Figure 2A, two negative cotton effects at 208 nm and 222 nm and one positive cotton effect at 195 nm were identified, indicating that BmADK had α-helix structures. The α-helix and β-strand contents were calculated to be 36.8% and 29.9%, respectively (http://dichroweb.cryst.bbk.ac.uk/html/process.shtml, last accessed date: 08 August 2018), which is consistent with our previous bioinformatic analysis (Figure 1A).

To illuminate the effects of temperature on BmADK secondary structure, various temperatures were tested. The thermal stability of BmADK was monitored based on θ_222_ shift in CD spectra. As shown in Figure 2B, the secondary structure of BmADK was stable from 10 to 30 °C, but the α-helix content of BmADK decreased at temperatures above 37.5 °C. The midpoint of the denaturation curve represents the melting temperature (Tm), which was calculated to be 46.51 ± 0.50 °C. The enthalpy and entropy changes (∆H and ∆S) were 253.43 ± 0.20 KJ/mol and 0.79 ± 0.01 KJ/(mol K), respectively.

The pH stability of BmADK was also monitored based on θ_222_ shift in CD spectra (Figure 2C). The results showed that its secondary structure was relatively stable in pH range of 5.0–11.0. When pH was lower than 5.0, the secondary structure of BmADK changed quickly.

### 2.3. Influence of Mutations on Enzymatic Activity

**Figure 3:**
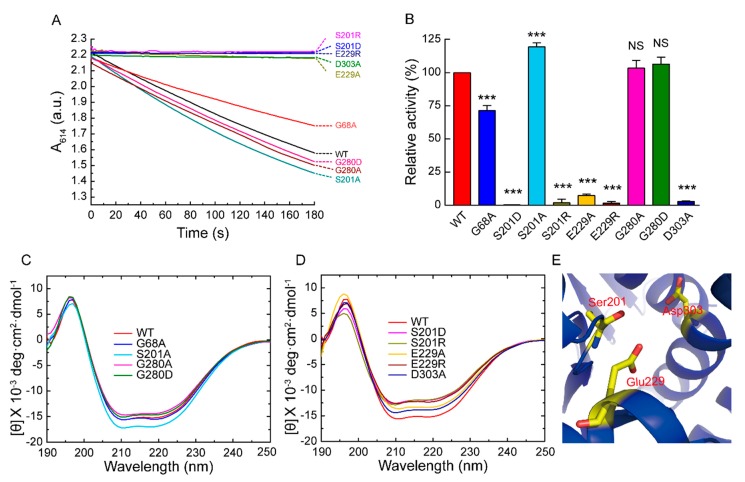
Influence of mutations on BmADK activity and secondary structure. (**A**) Determination of enzymatic activity of BmADK mutants and WT. (**B**) Comparison of enzymatic activity between BmADK mutants and WT. All experiments were performed in triplicate. Data are represented as mean ± SD. ***, *p* < 0.001, compared to the control. (**C**,**D**) CD spectra of BmADK and its mutants. The mean residue ellipticity was the arithmetic mean of three independent tests. (**E**) Homologous modeling of the substrate-binding pocket of BmADK. The catalytic residues (S201, E229, D303) are shown in sticks.

To further obtain the structural and activity information of BmADK, the site-directed mutagenesis of five conserved amino acid residues was performed. We obtained G68A, S201A, S201R, S201D, E229A, E229R, G280A, G280D, and D303A mutants and examined their enzymatic activity. Figure 3A shows a comparison of the BmADK mutants’ activity with that of WT BmADK. The change of absorbance at 614 nm (A_614_) showed that G68A, S201A, G280A, and G280D had similar effects on the absorbance decrease with that of WT; however, S201R, S201D, E229R, D303A, and E229A could not change the absorbance of the reaction system.

To reveal the mutations on the catalytic activity of BmADK, the relative activity of BmADK mutants was calculated with the enzymatic activity of WT as 100%, as shown in Figure 3B. The results showed that the catalytic activity of E229R, E229A, and D303A were completely lost. G280A and G280D had little effect on BmADK activity, but G68A resulted in a significant decrease in BmADK activity. Intriguingly, S201R and S201D completely abolished the activity of BmADK; however, S201A significantly enhanced BmADK enzymatic activity when compared with that of WT.

To explain the effect of the mutations on BmADK activity, the secondary structures of these mutants were analyzed by CD spectra. The results showed that the mutations had different effects on the secondary structure of BmADK. S201R, S201D, and E229R caused a significant decrease in the α-helix content of BmADK, whereas S201A increased the α-helix content of BmADK. E229A and D303A also resulted in a decrease in the α-helix content of BmADK (Figure 3C,D). G68A induced a tiny increase in the α-helix content of BmADK, whereas G280A and G280D resulted in a slight decrease in the α-helix content of BmADK (Figure 3C,D). Homologous modeling showed that S201, E229, and D303 were all located in the catalytic center of BmADK (Figure 3E), whereas G68 and G280 were away from the catalytic center of BmADK.

### 2.4. Expression Profile and Cellular Localization of BmADK

**Figure 4:**
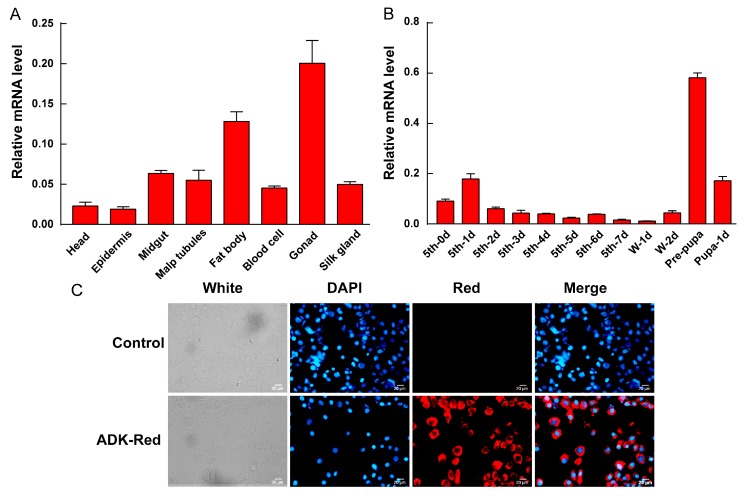
Expression profile and cellular location of BmADK. (**A**) Expression profile of *BmADK* in the head, malpighian tubules, gonad, midgut, fat body, silk gland, blood cell, and epidermis of the silkworm on the third day of the fifth instar. (**B**) Expression profile of *BmADK* in the silk gland at mRNA level from the first day of the fifth instar to the first day of the pupa of the silkworm. 5th, the fifth instar stage; W, wandering stage; Pupa, pupal stage. (**C**) Fluorescence location of BmADK in BmE cells. “Red” is the signal from recombinant BmADK. The cells were stained with DAPI for 20 min. Scale bar is 20 μm.

To reveal the potential role of *BmADK* on silkworm development, the expression profile of *BmADK* in different tissues was analyzed by quantitative real-time PCR (qRT-PCR). As shown in Figure 4A, *BmADK* was expressed in all tissues of the silkworm on the third day of the fifth instar. Notably, it was highly expressed in the fat body and gonads.

Furthermore, we analyzed the expression profile of *BmADK* in the silk gland from the first day of the fifth instar to the first day of pupa. qRT-PCR showed that the expression of *BmADK* increased from the fifth instar to day 1 of the fifth instar, then decreased to a low level until day 7 of the fifth instar. Afterward, *BmADK* expression increased from day 1 of the wandering stage, reached the maximum at the pre-pupal stage, then decreased on day 1 of the pupal stage (Figure 4B).

To understand the role of *BmADK* in cells, we constructed a 1180-BmADK-red eukaryotic expression vector, and then transfected it into BmE cells to analyze its cellular location. The red fluorescence signal indicated the location of BmADK-red in the cells (Figure 4C). Cell nuclei were dyed blue by DAPI. The signal, merged by red and blue, clearly indicated that BmADK was located in the cytoplasm of cells.

### 2.5. Effect of BmADK on Cell Apoptosis, Autophagy, and 20E Signaling

**Figure 5:**
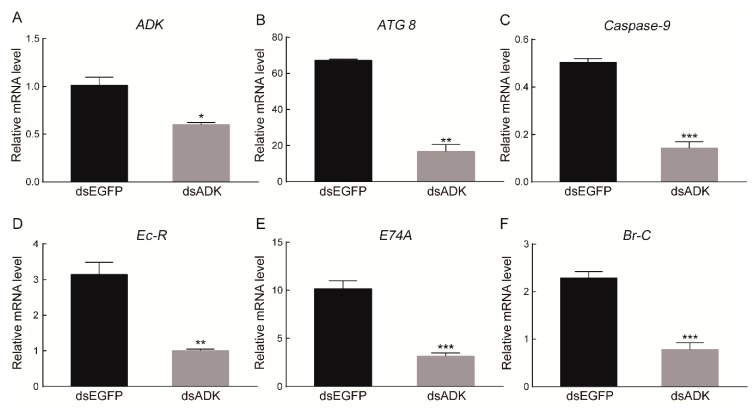
Effects of *BmADK* RNAi on cell apoptosis, autophagy, and 20E signaling. (**A**) *BmADK* expression at 36 h after RNAi with ds-BmADK. The expression of (**B**) *ATG 8*, (**C**) *Caspase-9*, (**D**) *Ec-R*, (**E**) *E74A*, and (**F**) *Br-C* at 36 h after RNAi with ds-BmADK. EGFP was used as the control. All experiments were performed in triplicate. Data are represented as mean ± SD. *, *p* < 0.05; **, *p* < 0.01; ***, *p* < 0.001, compared to the control.

To reveal the potential role of *BmADK*, we injected silkworm larvae on the seventh day of the fifth instar with ds-EGFP or ds-BmADK. After 36 h, a second injection was performed. qRT-PCR was performed to analyze the expression of *BmADK* and key genes of cell apoptosis and autophagy signaling related to silk gland degeneration. As shown in Figure 5A, the mRNA level of *BmADK* was downregulated after injection of ds-BmADK, indicating that double-stranded RNA interference (RNAi) of *BmADK* was successful. The expression of *ATG 8* and *Caspase-9* related to cell apoptosis and autophagy were also downregulated (Figure 5B-C). Meanwhile, the expression of *Br-C*, *Ec-R*, and *E74A* genes related to 20E signaling were also downregulated (Figure 5D–F).

## 3. Discussion

To date, although the three-dimensional structures of ADK from different species such as *Homo sapiens* [10], *Toxoplasma gondii* [13,19,20], *Mycobacterium tuberculosis* [14], *Anopheles gambiae* [21], *Trypanosoma brucei rhodesiense* [22], and *Schistosoma mansoni* [23] have been resolved, the biochemical properties, structure, and biological role of BmADK are largely unknown. In this work, we found that BmADK contained a large number of α-helix and β-strand structures which were similar to the secondary structures of ADK in other species [12,13,14]. The molecular weight of BmADK was 38 kDa, indicating that BmADK acted as a monomer in solution, while the active formation of MtADK is a functional homodimer [14]. The mutations did not alter the monomer state of BmADK in solution (Figure S1, Supplementary Materials).

The secondary structure of BmADK was stable in 10–30 °C (Figure 2B). Considering the important role of ADK in maintaining adenosine homeostasis, temperatures over 35 °C or below 10 °C are not suitable for sericulture, as too high or low temperature will change BmADK structure and activity, thus affecting silkworm growth and development. In brief, BmADK activity is pretty stable at ambient temperature, thus maintaining the normal physiological role of BmADK during silkworm development. The optimum temperature for silkworm rearing is around 25 °C, which is consistent with the optimal temperature of BmADK activity [24].

Two isoforms of ADK have been identified in various mammals, and they have no differences in biochemical and kinetic properties [3,4,25,26]. In mammal cells, a conserved motif (PKPKKLKVE) serves as the nuclear localization signal. Replacement of KK in this motif with either AD or AA abolishes its nuclear localization capability [27]. Here, only one form of BmADK was identified in the silkworm genome base, which is different from ADKs in mammalian ADKs. Moreover, the similar motif in mammalian ADKs responding for nuclear localization signal could not be found in the sequence of BmADK, indicating that BmADK may not be detected in the cellular nuclei. Our result showed BmADK was located in the cytoplasm of cells (Figure 4C), which is consistent with our previous prediction from the sequence comparison. Unlike the cellular location of mammalian ADKs in the nucleus, therefore, BmADK is not likely involved in the process of methyl transfer [15].

The mutations of G280A and G280D had no significant influence on the enzymatic activity compared with that of WT, indicating that it was not a key amino acid for enzymatic activity. CD analysis showed G280A and G280D had similar secondary structures with that of WT (Figure 3C). G68 and G69 of BmADK (G63 and G64 of human ADK) are conserved in different organisms. According to human ADK structure, the big domain and the small lid-like domain are connected by four loops as the hinge, which create the reaction cavity for adenosine. The conformation of the catalytic center is further stabilized by adenosine molecules [10,12]. The activity of G68A was approximately 70% of BmADK activity (Figure 3B), implying that G68 of BmADK, like G63 of human ADK, was also located in the hinge. The mutation of G68A induced a slight increase in the α-helix content of BmADK (Figure 3C), which likely affected the interaction between the big domain and the small lid-like domain, thus leading to conformational change of the catalytic center. Although G68A mutation reduced BmADK activity, it did not cause complete inactivation of BmADK since glycine (G) and alanine (A) are very similar in structure.

In human ADK, S198, E226, and D300 (S201, E229, and D303 of BmADK) are involved in substrate binding and catalytic reaction [10,12]. D300 is an important catalytic residue which bridges Mg^2+^ and O5′-hydroxyl of adenosine. In the catalytic reaction, the O5′-hydroxyl donates a hydrogen bond to D300 and accepts a hydrogen bond from a water molecule, which forms a hydrogen bond to another water molecule. This water molecule is a part of the Mg^2+^ coordination sphere [12]. D303A mutation resulted in the decrease in the α-helix content of BmADK and the loss of BmADK activity (Figure 3B,D), which likely alters the conformation of the catalytic center and prevents alanine from acting as an acceptor of hydrogen bonds, thus affecting the catalytic reaction. E226 is involved in ADP/ATP binding and coordinating Mg^2+^ and absolutely conserved in different species [12]. Therefore, the mutations of E229A and E229R prevent alanine (A) and arginine (R) from forming proper hydrogen bonds with the substrate. In addition, E229A and E229R changed the α-helix content of BmADK (Figure 3D), thus affecting the conformation of the catalytic center (Figure 3E). As a result, the enzyme was deactivated completely by the mutations of E229A and E229R (Figure 3B). The α-helix content of E229R was less than that of E229A. The possible reason is that R has a negative charge side group, whereas E has a positive charge side group. The opposite charge mutation (E->R) changes the environment more significantly than the mutation E->A, thus further resulting in the decrease in the α-helix content and catalytic activity of BmADK.

Intriguingly, the mutants S201A, S201D, and S201R showed different activity. S201R and S201D completely abolished BmADK activity; however, S201A significantly enhanced its enzymatic activity when compared with that of WT (Figure 3B). Compared to serine, arginine (R) and aspartic acid (D) have a longer charged R-group. The R-group of alanine (A) is shorter than that of serine and lacks a polar hydroxyl group. CD spectra showed that the α-helix content of S201A was significantly increased when compared to that of WT (Figure 3C). Thus, the increase in S201A activity suggested that the mutation of S->A may ensure the stability of the reaction center conformation and provide a favorable environment for the binding of substrate and the reaction. On the contrary, the α-helix contents of S201D and S201R were significantly decreased when compared to that of WT (Figure 3D). As a result, the activity loss of S201D and S201R could be ascribed to the mutations of S->D and S->R, respectively, which may disrupt the stability of the catalytic center conformation and change its conformation. Additionally, the replacement of the hydroxyl group (S) with a long-chain and charged R-group (D/R) could form steric hindrance, which is not favorable for the entrance and binding of substrate. Our results suggested that S201 is a key amino acid for the enzymatic activity of BmADK. The analysis on BmADK mutants provides us more knowledge about the relationship of ADK activity and its structure. In particular, the increase in S201A activity directs a new approach to regulate ADK activity and explore its biological role related to ADK dysfunction, disease, and cancer.

*BmADK* was highly expressed in the silk glands at the pre-pupal stage (Figure 4B), indicating that it might be related to the metamorphosis of silkworm larvae into the pupa. At this stage, cell apoptosis and autophagy occur in silk gland cells to regulate silk gland degeneration [28]. RNAi indicated that the downregulation of *BmADK* led to the downregulation of *ATG 8* and *Caspase-9* related to cell apoptosis and autophagy (Figure 5A–C), suggesting that *BmADK* may be involved in cell apoptosis and autophagy of silk glands. In fruit flies and silkworm, the degradation of intestines and silk glands is regulated by 20E signaling [29,30]. qRT-PCR showed that the expression of *Br-C*, *Ec-R*, and *E74A* genes related to 20E signaling was significantly reduced after RNAi of *BmADK* (Figure 5D–F), indicating that *BmADK* is likely associated with 20E signaling pathway. Our results suggested that *BmADK* is likely involved in 20E signaling pathway, cell apoptosis, and autophagy during the pre-pupal stage of silkworm, thus regulating silk gland degeneration and silkworm metamorphosis. This novel role of *BmADK* in silkworm is different from previous reports of mammalian ADKs including regulating methylation pathway [15], promoting t-lymphocytes proliferation [16] and inflammation [17], and promoting liver cancer cell proliferation [18], which will advance a better understanding of ADK and its biological role.

It was noted that although silencing of *BmADK* was significant, more than half of the transcript was still present and not silenced (Figure 5A). Therefore, BmADK was most likely still produced, although to a lower amount. As a result, the transcriptions of *ATG 8*, *Caspase-9*, *Ec-R*, *E74A*, and *Br-C* were downregulated temporarily, and then recovered to normal levels after the disappearance of the RNAi effect. Such transient downregulation of *ATG 8*, *Caspase-9*, *Ec-R*, *E74A*, and *Br-C* may not be powerful to affect cell apoptosis and autophagy. Hence, it was difficult to observe the difference of silk gland degeneration between the wild-type and RNAi silkworm.

## 4. Materials and Methods

### 4.1. Chemicals and Materials

The silkworm strain Dazao, used in this study, was provided by the State Key Laboratory of Silkworm Genome Biology, Southwest University, Chongqing, China. BmE cell line was derived from the embryonic cells of the silkworm and reserved in our laboratory. The plasmid DNA extraction kit was purchased from TransGene (Beijing, China). The reverse transcription kit was obtained from Promega (Madison, WI, USA). The transfection reagent was a product of Roche (Basel, Switzerland). The specific primers were synthesized by Sangon Biotech (Shanghai, China). Polyoxymethylene was obtained from Sangon Biotech (Shanghai, China). Anti-fluorescence quenching agent and DAPI were obtained from Beyotime (Beijing, China). The prokaryotic expression plasmid pSKB2 was a gift from Prof. Xuewu Zhang (University of Texas Southwestern Medical Center at Dallas, TX, USA) and stored in our laboratory. The restriction enzymes were products of Takara (Tokyo, Japan).

### 4.2. Bioinformatic Analysis

BmADK sequences was obtained from the National Center for Biotechnology Information. BLAST was performed using the online server (https://blast.ncbi.nlm.nih.gov/Blast.cgi, last accessed date: 18 August 2018). Multiple sequence alignment was performed using the ClustalW algorithm [31]. The secondary structure of BmADK was predicted using PSIPRED (http://bioinf.cs.ucl.ac.uk/psipred/, last accessed date: 18 June 2018). The three-dimensional structure of BmADK was predicted on the I-TASSER server [32,33]. PyMOL (ver 0.99, DeLano Scientific LLC, Palo Alto, CA, USA) were used to render the BmADK structure.

### 4.3. Cloning, Expression, and Purification of BmADK and Its Variants

The *BmADK* gene was amplified by PCR using *Bombyx mori* (Dazao) silk gland cDNA library as the template, using specific primers (ADK-F: CGCGGATCCATGGACGTTTCTGATTCCATATGTG, ADK-R: CCCAAGCTTTCAGTCATTGTATTCGCTGGGTC). The PCR product was gel purified, recovered, and ligated with a pET28a-derived vector pSKB2. The plasmids for G68A, S201A, S201R, S201D, E229A, E229R, G280A, G280D, and D303A mutants were generated by the Agilent QuikChange site-directed mutagenesis kit (Santa Clara, CA, USA). The primer sets for BmADK mutants are shown in Table S1 (Supplementary Materials). The integrity of the sequence was confirmed by DNA sequencing. BmADK was expressed in *Escherichia coli* BL21 (DE3) strain. The expression of BmADK was induced by 0.1 mM isopropyl β-D-1-thiogalactopyranoside (IPTG) at 25 °C for 10 h when OD_600_ reached 0.6. Cells were harvested by centrifugation at 6,000× *g* for 20 min, and then resuspended in lysis buffer (20 mM Tris-HCl, pH 8.0, 500 mM NaCl, 20 mM imidazole, 5% glycerol). After sonication (7.5 min), the supernatant was collected by centrifugation at 15,000× *g* for 15 min, and then loaded onto a Ni-NTA affinity column (GE Healthcare, Chicago, IL, USA) equilibrated with binding buffer (20 mM Tris-HCl, pH 8.0, 500 mM NaCl, 20 mM imidazole, 5% glycerol). Subsequently, the target protein was eluted with elution buffer containing 100 mM imidazole. After concentration by ultrafiltration, BmADK was loaded onto a HiLoad 16/60 Superdex 75 column (GE Healthcare, Chicago, IL, USA) equilibrated with 20 mM Tris-HCl, pH 8.0, 50 mM NaCl, 5% glycerol. The purity of recombinant BmADK was estimated by 12% SDS-PAGE. The purified protein was concentrated to 3.68 mg/mL and stored at –80 °C. Protein concentration was determined using the extinction coefficient of 24,870 M^−1^·L·cm^−1^ at 280 nm on a NanoDrop 2000C spectrophotometer (Thermo Fisher, Waltham, MA, USA). BmADK variants were expressed, purified, and stored in the same manner as the WT protein.

### 4.4. Analysis of Enzymatic Activity

The enzyme activity was analyzed by spectrophotometric assay which uses bromothymol blue as a pH indicator, and the absorbance change is detected at 614 nm [24]. The reaction mixture had a total volume of 20 mL including 2 mL 0.1% bromothymol blue, 1 mL 1 M Gly-NaOH (pH 9.0), 1 mL 0.1 M magnesium acetate (MgAC_2_), 48 mg ATP, and 53 mg adenosine. The initial absorbance of the freshly prepared reaction mixture at 614 nm was adjusted to 2.2 with 0.5 M NaOH appropriately. The reaction was triggered by the addition of BmADK (5 μL, 3.68 mg/mL) into the reaction mixture (995 μL). The absorbance of the reaction system was recorded at 614 nm within 180 s on a DU800 nucleic acid/protein analyzer (Beckman, Brea, CA, USA) at 25 °C using a plastic cuvette with a light path of 1 cm. Each test was repeated in triplicate. One-way analysis of variance was used for the significance analysis.

### 4.5. CD Spectroscopy

CD spectra were collected on a MOS-500 CD spectrometer (BioLogic, Seyssinet-Pariset, France) with a 0.05 cm quartz cuvette at 25 °C in 190–250 nm using standard procedures. BmADK was dissolved in 20 mM Tris-HCl, 150 mM NaCl, pH 8.0 buffer to 0.345 mg/mL. The effects of pH on the secondary structure of BmADK were recorded in the pH range of 3.0–11.0. The thermal denaturation of BmADK was performed from 10 to 87.5 °C at an incremental step of 2.5 °C. The mean residue ellipticity at 222 nm was used to characterize the structural changes of BmADK induced by temperature and pH after subtraction of baseline corrections. Each test was repeated in triplicate. The mean residue ellipticity was the arithmetic mean of three independent tests.

### 4.6. Cellular Location

The cellular location of BmADK was determined as described by Liu [34]. BmE cells were grown on glass coverslips. The recombinant eukaryotic over-expression vector 1180-BmADK-red was transfected into BmE cells using X-tremeGENE HP DNA transfection reagent. After 48 h, cells were washed thrice with PBS buffer, and then fixed in 4% (*w*/*v*) paraformaldehyde (PFA) for 15 min at 25 °C. Then, BmE cells were stained with 9,6-diamidino-2-phenylindole (DAPI, 1:10,000) for 15 min after washing three times with PBS. Afterward, the cells were washed thrice with PBS. The coverslips were mounted on glass slides and sealed with anti-fluorescence quenching reagent (Beyotime, Beijing, China). Fluorescence signals were observed on a confocal fluorescence microscopy Fv1000 (Olympus, Tokyo, Japan). Each test was repeated in triplicate.

### 4.7. Expression Profile of BmADK

The silkworm strain Dazao was reared on fresh mulberry leaves at room temperature with 70 ± 5% humidity in our laboratory. Head, malpighian tubules, gonad, midgut, fat body, silk gland, blood cell, and epidermis were dissected from silkworm larvae on the third day of the fifth instar. Silk glands were dissected from silkworm larvae from the first day of the fifth instar to the first day of silkworm pupa. All tissues were immersed in PBS buffer, washed three times, and then immediately frozen in liquid nitrogen and stored at −80 °C for RNA isolation or protein extraction.

qRT-PCR was performed to analyze *BmADK* expression in different tissues on the third day of the fifth instar larvae or at different periods in silk gland. The specific primers for qRT-PCR were designed using Primer Premier 5.0 (BmADK-F: AAGAAGGATTATTAGTGGGC, BmADK-R: ACGAGTTCCGAGTAGAGTGG). Total RNA was extracted using TRIzol Reagent (Invitrogen, Carlsbad, CA, USA). The cDNA was prepared using a reverse transcription kit from Promega. The products were amplified and detected on a Life 7500 FAST Real-Time PCR System (Applied Biosystems, Foster City, CA, USA) using SYBR Premix Ex Taq kit (Takara, Kyoto, Japan) as according to the manufacturer’s protocol. The reaction mixture (20 µL) was performed as follows: denaturation at 95 °C for 30 s followed by 40 cycles at 95 °C for 5 s and 60 °C for 30 s. The endogenous glyceraldehyde-3-phosphate dehydrogenase of the silkworm (BmGAPDH) was used as a control. All tests were performed in triplicate. The significance was analyzed by one-way analysis of variance.

### 4.8. Double-Stranded RNA Interference (RNAi)

The template DNAs of *BmADK* and EGFP (control) used for double-stranded RNA (dsRNA) synthesis were amplified by PCR using primers (BmADK-F: GTAATACGACTCACTATAGGGCG ATGTGCAGGAGATCGCTTTA, BmADK-R: GTAATACGACTCACTATAGGGTCAGTCATTGTA TTCGCTGGGTCCG), and purified using a gel extraction kit (Omega, Norcross, GA, USA). Double-stranded RNAs were synthesized using a Promega RiboMAX large scale RNA production system-T7 as according to the manufacturer’s instructions and assessed by 1.2% agarose gel. The concentration of double-stranded RNAs was measured on a Thermo Fisher NanoDrop 2000C spectrophotometer. Silkworm larvae on day 7 of the fifth instar were injected with double-stranded EGFP (dsEGFP) and double-stranded BmADK (dsBmADK), respectively. DsRNA (50 μg) was injected into the hemolymph of silkworm larvae through the spiracle using a capillary needle. After 36 h, a second injection was carried out. Total RNA was prepared from the silk gland of silkworm larvae at the pre-pupal stage with Invitrogen TRIzol Reagent. All experiments were performed in three independent replicates, and the significance was analyzed as the previous method. Relative expression levels of *BmADK*, *ATG 8*, *Caspase-9*, *Ec-R*, *E74A*, and *Br-C* were measured at 48 h after injection [35].

## 5. Conclusions

In summary, BmADK is a monomer in solution, and contains 36.8% α-helix and 29.9% β-strand structures, respectively. The secondary structure of BmADK is stable in pH 5.0–11.0, and almost not affected under 30 °C. G68, S201, E229, and D303 are key amino acids for BmADK structure and activity. S201A mutation significantly increases the α-helix content of BmADK and its enzymatic activity. BmADK is located in the cytoplasm of cells. BmADK is likely involved in 20E signaling, apoptosis, and autophagy of silkworm by regulating the expression of *ATG 8*, *Caspase-9*, *Br-C*, *Ec-R*, and *E74A*.

## Supplementary Materials

Supplementary materials can be found at https://www.mdpi.com/1422-0067/20/15/3732/s1.
