# Supplementary material for: A Novel Adenosine Kinase from Bombyx mori: Enzymatic Activity, Structure, and Biological Function
Source: Int J Mol Sci. 2019 Jul 31;20(15):3732. doi: 10.3390/ijms20153732 (PMC6695918; doi:10.3390/ijms20153732)
Supplement: Supplementary file 1 [file ijms-20-03732-s001.zip › ijms-555986-SI.pdf]

Table S1. The primer sets of BmADK mutants.

Name	Sequences
G68A-F	GCAGAGTATATTGCTGCCGGAAGCGTTCAG
G68A-R	CTGAACGCTTCCGGCAGCAATATACTCTGC
S201A-F	GTTATGAACTTGGCCGCGCCCTTTGTCTC
S201A-R	GAGACAAAGGGCGCGGCCAAGTTCATAAC
S201D-F	GTTATGAACTTGGATGCGCCCTTTGTCTC
S201D-R	GAGACAAAGGGCGCATCCAAGTTCATAAC
S201R-F	GTTATGAACTTGCGCGCGCCCTTTGTCTC
S201R-R	GAGACAAAGGGCGCGCGCAAGTTCATAAC
E229R-F	CGGGAATGAATCGCGCGCAGACGCTTTCG
E229R-R	CGAAAGCGTCTGCGCGCGATTCATTCCCG
E229A-F	CGGGAATGAATCGGCCGCAGACGCTTTCG
E229A-R	CGAAAGCGTCTGCGGCCGATTCATTCCCG
G280A-F	TGGTACAGAGCGCCCGAGTCACCCTGATA
G280A-R	TATCAGGGTGACTCGGGCGCTCTGTACCA
G280D-F	TGGTACAGAGCGATCGAGTCACCCTGATA
G280D-R	TATCAGGGTGACTCGATCGCTCTGTACCA
D303A-F	ACAAACGGTGCCGGAGCCGCCTTTACTGGT
D303A-R	ACCAGTAAAGGCGGCTCCGGCACCGTTTGT

Figure S1

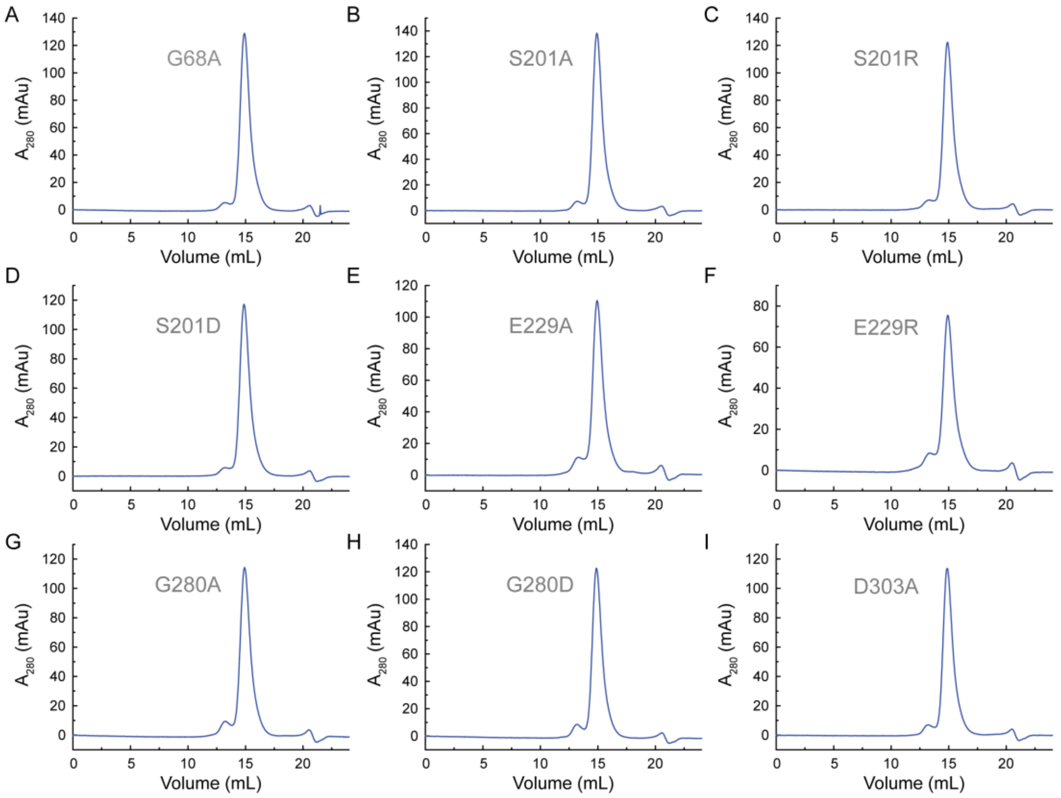

Figure S1. Purification of BmADK mutants by gel filtration.
